# Dengue and Other Common Causes of Acute Febrile Illness in Asia: An Active Surveillance Study in Children

**DOI:** 10.1371/journal.pntd.0002331

**Published:** 2013-07-25

**Authors:** Maria Rosario Capeding, Mary Noreen Chua, Sri Rezeki Hadinegoro, Ismail I. H. M. Hussain, Revathy Nallusamy, Punnee Pitisuttithum, Kusnandi Rusmil, Usa Thisyakorn, Stephen J. Thomas, Ngoc Huu Tran, Dewa Nyoman Wirawan, In-Kyu Yoon, Alain Bouckenooghe, Yanee Hutagalung, Thelma Laot, Tram Anh Wartel

**Affiliations:** 1 Research Institute for Tropical Medicine, Muntinlupa City, Philippines; 2 Chong Hua Hospital, Cebu City, Philippines; 3 Medical Faculty, University of Indonesia, Jakarta, Indonesia; 4 Hospital Kuala Lumpur, Kuala Lumpur, Malaysia; 5 Penang Hospital, Penang, Malaysia; 6 Faculty of Tropical Medicine, Mahidol University, Bangkok, Thailand; 7 Department of Child Health, Hasan Sadikin Hospital/School of Medicine Padjadjaran University, Bandung, Indonesia; 8 Armed Forces Research Institute of Medical Sciences (AFRIMS), Bangkok, Thailand; 9 Ho Chi Minh City Pasteur Institute, Ho Chi Minh City, Vietnam; 10 School of Medicine, Udayana University, Bali, Indonesia; 11 Sanofi Pasteur, Clinical Research and Development, Singapore; 12 Sanofi Pasteur, Clinical Research and Development, Makati City, Philippines; 13 Sanofi Pasteur, Clinical Research and Development, Bangkok, Thailand; Institute of Tropical Medicine, Belgium

## Abstract

**Background:**

Common causes of acute febrile illness in tropical countries have similar symptoms, which often mimic those of dengue. Accurate clinical diagnosis can be difficult without laboratory confirmation and disease burden is generally under-reported. Accurate, population-based, laboratory-confirmed incidence data on dengue and other causes of acute fever in dengue-endemic Asian countries are needed.

**Methods and principal findings:**

This prospective, multicenter, active fever surveillance, cohort study was conducted in selected centers in Indonesia, Malaysia, Philippines, Thailand and Vietnam to determine the incidence density of acute febrile episodes (≥38°C for ≥2 days) in 1,500 healthy children aged 2–14 years, followed for a mean 237 days. Causes of fever were assessed by testing acute and convalescent sera from febrile participants for dengue, chikungunya, hepatitis A, influenza A, leptospirosis, rickettsia, and *Salmonella* Typhi. Overall, 289 participants had acute fever, an incidence density of 33.6 per 100 person-years (95% CI: 30.0; 37.8); 57% were IgM-positive for at least one of these diseases. The most common causes of fever by IgM ELISA were chikungunya (in 35.0% of in febrile participants) and *S.* Typhi (in 29.4%). The overall incidence density of dengue per 100 person-years was 3.4 by nonstructural protein 1 (NS1) antigen positivity (95% CI: 2.4; 4.8) and 7.3 (95% CI: 5.7; 9.2) by serology. Dengue was diagnosed in 11.4% (95% CI: 8.0; 15.7) and 23.9% (95% CI: 19.1; 29.2) of febrile participants by NS1 positivity and serology, respectively. Of the febrile episodes not clinically diagnosed as dengue, 5.3% were dengue-positive by NS1 antigen testing and 16.0% were dengue-positive by serology.

**Conclusions:**

During the study period, the most common identified causes of pediatric acute febrile illness among the seven tested for were chikungunya, *S.* Typhi and dengue. Not all dengue cases were clinically diagnosed; laboratory confirmation is essential to refine disease burden estimates.

## Author Summary

Acute febrile episodes are common in children living in tropical countries. Diagnosis can be challenging because symptoms of the more common infectious causes are similar and often mimic those of dengue. Asia Pacific has over 70% of the worldwide dengue disease burden, although dengue incidence is generally underestimated because most surveillance systems are passive or based on clinical diagnosis without laboratory confirmation. Understanding the local etiology of febrile illness and the incidence of dengue is important when planning large-scale vaccine trials. This prospective, active fever surveillance, cohort study was carried out in children in five dengue-endemic Asian countries – Indonesia, Malaysia, Philippines, Thailand and Vietnam – during 2010–2011. Acute febrile episodes occurred in 289 (19.3%) of the cohort of 1,500 children. Among the diseases for which antibodies were tested using commercial kits, the top three causes of acute fever were chikungunya, *Salmonella* Typhi and dengue, followed by influenza A, rickettsia and hepatitis A. Dengue was confirmed in 11.4% of the febrile children by viral protein detection and in 23.9% by serology. Clinical diagnosis was not sufficient to detect all dengue cases. These findings are of relevance to those planning clinical studies of vaccines against these infectious agents in Southeast Asia.

## Introduction

Undifferentiated febrile illnesses are common in children living in tropical areas of Asia. Common causes include dengue, malaria, leptospirosis, influenza A, *Salmonella* Typhi, rickettsia, Japanese encephalitis and chikungunya, [1]–[8]. The symptoms and differential diagnoses of these diseases are similar, often mimicking those of dengue and making accurate clinical diagnosis difficult without laboratory confirmation [1], [9]. Reliable laboratory-confirmed diagnoses of acute febrile illness require a positive bacteriological/virological test such as culture results and PCR; serological confirmation of pathogen-specific antibodies (immunoglobulin (Ig)M or a four-fold rise in IgG) can also support such assessments.

Dengue is caused by four serotypes (DEN1–4) of the genus Flavivirus [10]. Transmitted by *Aedes* mosquitoes, it is one of the most widespread of the arthropod-borne viral diseases. It is a major public health concern because of the huge burden it exerts on populations, health systems and economies [11]. Asian-Pacific countries have more than 70% of the worldwide disease burden [12], and in Indonesia and Thailand, dengue is one of the leading causes of hospitalization and death among children [13].

Although dengue prevention currently relies on mosquito control, vaccine candidates are under development [5], [14], [15] and the World Health Organization (WHO) has included dengue among its targets for the control of neglected tropical diseases during 2015–2020 [11]. However, the burden of dengue is generally under-reported in many Asian countries, because national surveillance systems (where they exist) are passive and/or based largely on clinical diagnosis without laboratory confirmation [16]–[19]. Thus, there is a need for accurate, population-based, laboratory-confirmed data on the incidence of dengue in high-risk populations. Determining the local etiology of acute febrile illness and the operational suitability of field sites in endemic regions is also important for the success of large-scale clinical trials of dengue vaccines.

This prospective cohort study in children was therefore carried out in five dengue-endemic countries: Indonesia, Malaysia, Philippines, Thailand and Vietnam. Active surveillance for febrile illness was carried out in the cohort population to determine the incidence and proportion of acute febrile episodes that were caused by dengue, as well as by chikungunya, hepatitis A and influenza A viruses, leptospirosis, rickettsia, and *S.* Typhi. These non-dengue diseases were chosen because they are the more frequently reported causes of febrile illness caused by a single pathogenic organism at the study sites and because their differential diagnoses mimic that for dengue [9].

## Methods

### Ethics statement

The study protocol was approved by the site-specific Independent Ethics Committee or Institutional Review Board (IRB); namely the Committee of Medical Research Ethics, Faculty of Medicine, University of Indonesia; the Health Research Ethics Committee, Faculty of Medicine, University Padjadjaran/Dr Hasan Sadikin Hospital; Faculty of Medicine Udayana University/Sanglah Hospital Ethics Committee, Bali, Indonesia; the Medical Research & Ethics Committee, Ministry of Health Malaysia; Research Institute for Tropical Medicine IRB, Philippines; Chong Hua Hospital IRB and the Vicente Sotto Memorial Medical Center Ethics Committee, Cebu, Philippines; the Walter Reed Army Institute of Research IRB, US (Kamphaeng Phet Hospital) and the Philippines (Cebu); the Ethical Review Committee for Research in Human Subjects, Ministry of Public Health, Thailand; the Ethics Committee, Faculty of Tropical Medicine, Mahidol University; and the Biomedical Research Ethics Committee, Ministry of Health, Vietnam.

The study was conducted in accordance with the Seoul revision of the Declaration of Helsinki as adopted by the concerned regulatory authorities, using Good Clinical Practice and International Conference on Harmonization guidelines.

Before any procedure associated with the study was performed, parents/guardians provided written informed consent on behalf of all child participants. In addition, written consent was also obtained through separate assent forms from participants according to local Ethics Committee regulation requirements or according to the study sponsor's standard operating procedures in countries that do not have local requirements for assent forms (Indonesia, Malaysia, Thailand: 7–14 years ; Philippines: 12–14 years; Vietnam: 8–11 years). Furthermore, participants aged 12–14 years in Vietnam also provided signed informed consent on the same consent form as their parents/guardians.

### Study sites

The study was conducted at 10 main sites in five Asian countries. These included district, city and provincial government hospitals and institutions in highly dengue-endemic areas, and associated health centers (satellite sites).

#### Indonesia

Cipto Mangunkusumo General Hospital in Jakarta; Child Health Department of Hasan Sadikin Hospital, Bandung, West Java and three satellite health centers (Garuda Health Center, Puter Health Center and Ibrahim Adjie Health Center); and Sanglah Hospital, Bali.

#### Malaysia

Hospital Kuala Lumpur and three satellite health clinics (Batu, Jinjang and Putrajaya); and Penang General Hospital.

#### Philippines

Governmental healthcare facilities, namely City Health Office and Del Remedio Health Center in San Pablo City and Research Institute for Tropical Medicine, Muntinlupa City; and in Barangay Guadalupe, Cebu City, the Guadalupe Health Center, Vicente Sotto Memorial Medical Center, Chong Hua Hospital and Philippines-AFRIMS Virological Research Unit.

#### Thailand

Ban Pong and Potharam hospitals, Ratchaburi province; and Kamphaeng Phet Provincial hospital, Kamphaeng Phet province, in collaboration with Mahidol University, Faculty of Tropical Medicine and the Kamphaeng Phet Armed Forces Research Institute of Medical Sciences (AFRIMS) Virology Research Unit.

#### Vietnam

Tien Giang General Hospital, My Tho City, Tien Giang Province.

### Study design

This prospective, multicenter, active surveillance cohort study conducted in Indonesia, Malaysia, Philippines, Thailand and Vietnam involved 150 participants from each main site, who were aged 2–14 years on the day of enrollment and recruited between June and September 2010. The study was conducted from June 2010 to July 2011.

Participants were recruited from the community, schools, health centers and/or private health clinics, depending on each study site's setting. Because one objective of this study was site preparation for a subsequent Phase III study of a dengue vaccine, eligibility criteria were established: namely, that participants had to be in good health with no history of chronic illness or immunodeficiency; able to attend scheduled visits and comply with study procedures; and had not received any vaccine in the 4 weeks preceding the day of enrollment (except for pandemic influenza vaccination, which could be received >2 weeks before enrollment), nor were planning to receive any vaccine in the 4 weeks following enrollment.

The active surveillance system was designed to detect all acute febrile episodes in the cohort. Participants' guardians were given a thermometer and shown how to measure axillary temperature. All participants or their guardians were contacted weekly to monitor the occurrence of acute febrile episodes. In the event of an episode, participants were asked to go to their designated healthcare center. To determine whether participants had to be contacted and followed up, school registers were monitored for absenteeism.

All participants made two visits to the study site: an enrollment visit and a termination visit. Additional visits were required if acute febrile episodes occurred: an acute visit and a convalescent visit, at which blood samples were obtained. Causes of fever were assessed by testing acute and convalescent sera for dengue, chikungunya, hepatitis A, influenza A, leptospirosis, rickettsia, and *S.* Typhi using the same standardized commercial kits at all sites. Malaria was not tested for because, based on the investigators' experience, malaria was not a significant cause of pediatric acute febrile illness at these study sites.

### Objectives, outcome assessment methods and case definitions

The study objectives were to identify acute febrile episodes among the cohort, and then to determine some of the specific causes of the acute fever in these febrile participants using a preset list of laboratory tests. A secondary objective was to evaluate operational infrastructure at these study sites in preparation for a Phase III study of a dengue vaccine [20].

The primary outcome measures were the proportion and incidences of acute febrile episodes, and which of the seven diseased tested for were their most common causes, based on the following case definitions.

#### Acute febrile episode

At least 2 consecutive days of fever (≥38°C). Consecutive febrile episodes separated by a symptom-free (i.e. fever-free) interval of more than 14 days were regarded as separate episodes.

#### Clinical dengue diagnosis

When febrile participants presented at the study sites, the attending clinicians were asked to specify whether or not the child had dengue, according to the 1997 WHO dengue case definition [21], i.e. an acute febrile illness with two or more of the following manifestations: headache, retro-orbital pain, myalgia, arthralgia, rash, hemorrhagic manifestations, leukopenia. Dengue hemorrhagic fever was classified as Grade I: fever accompanied by non-specific constitutional symptoms, positive tourniquet test and/or easy bruising; Grade II: spontaneous bleeding in addition to manifestations of Grade I patients, usually in forms of skin or other hemorrhages; Grade III: circulatory failure manifested by a rapid, weak pulse and narrowing of pulse pressure or hypotension with the presence of cold, clammy skin and restlessness; and Grade IV: profound shock with undetectable blood pressure or pulse.

#### Laboratory-confirmed dengue

Detection of dengue in the acute serum sample by nonstructural (NS) protein 1 enzyme-linked immunosorbent assay (ELISA) antigen test (virological confirmation).

#### Probable dengue

Based on serological criteria; i.e. IgM was observed in the acute or in the convalescent sample and/or a fourfold increase in IgG was observed between the acute and the convalescent samples (serological confirmation).

#### Other causes of fever

These were assessed using a panel of commercially available serological tests for chikungunya, hepatitis A, influenza A, leptospirosis, rickettsia, and *S.* Typhi. To avoid the confounding factors introduced in cases where previous infections could have resulted in a positive IgG result, only IgM data are reported here as evidence for the cause of acute fever.

Attending clinicians at participating sites also made a clinical diagnosis based on presenting signs and symptoms so that the participants could be managed appropriately according to local standard practice while laboratory tests were being processed. Although they were asked to specify an ‘Other diagnosis’ (in addition to dengue) for data capture purposes, information on case management and outcome were not collected during the study.

### Sample collection and data management

For every acute febrile episode, blood samples for acute sera were taken from the participants at the study site within 5 days after fever onset, and convalescent paired samples were obtained 7–14 days after acute sample collection for serological dengue tests, complete blood count (including platelet count and hematocrit), and to assess for other causes of febrile illness. Dengue NS1 tests were performed only on acute sera.

Clinical study information gathered at each study site was electronically reported by the study investigator or an authorized designee using an electronic case report form.

### Laboratory methods

The same commercial kits were used at each study site.

The Platelia Dengue NS1 Ag kit (Bio-Rad, USA) was used according to the manufacturer's instructions to detect dengue NS1 antigen in acute serum samples by ELISA. The Dengue Virus IgM Capture DxSelect ELISA kit and the Dengue Virus IgG Capture DxSelect ELISA kit (Focus Diagnostics, USA) were used according to the manufacturer's instructions to detect dengue-specific IgM or IgG, respectively, in both acute and convalescent samples.

Other causes of fever were assessed using commercial kits to detect leptospirosis (Leptospirosis Indirect Hemagglutination (IHA) Test; Focus Diagnostics, USA); rickettsia (Rickettsia IFA IgG/IgM; Focus Diagnostics, USA); hepatitis A (Anti-HAV IgM ELISA; DIAsource ImmunoAssays S.A., Belgium); *S.* Typhi (*Salmonella* Typhi IgM ELISA; Calbiotech Inc, USA); chikungunya (NovaLisa Chikungunya IgM μ-capture ELISA; NovaTec Immundiagnostica GmbH, Germany) and influenza A (NovaLisa Influenza Virus A IgM-ELISA; NovaTec Immundiagnostica GmbH, Germany) in both acute and convalescent sera. These kits were provided to all the sites for reasons of availability, ease of use and consistency, even though they were not all gold standard tests. The sensitivity and specificity of these tests, as determined by the manufacturers, are shown in Table S1.

### Statistical analyses

The sample size of 150 participants per site was not hypothesis-driven, and was based on an estimated proportion of acute febrile episodes in these locations of 24%, in accordance with the investigators' experiences.

The incidence and proportion of acute febrile episodes and their causes were described for the study cohort by country and for all countries combined. The Clopper-Pearson method was used to calculate the 95% confidence interval (CI) for the proportions of acute febrile illness and dengue [22]. The incidence density of acute febrile illness and of the causes was calculated as: 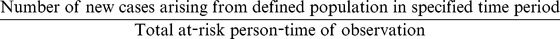
(2)


The Rothman-Greenland method [23] was used to calculate the CI of incidence density.

Statistical analyses were performed using SAS 9.1 software (SAS Institute Inc.). Missing data were not imputed.

## Results

### Study cohort, demographics and study duration

The study cohort included 1,500 eligible participants, of which 1,487 (99.1%) participants (446 [99.1%], 299 [99.7%], 297 [99.0%], 299 [99.7%] and 146 [97.3%] in Indonesia, Malaysia, Philippines, Thailand and Vietnam, respectively) completed the study. Demographic characteristics of the participants are shown in Table 1. Of the 13 participants (0.9%) who did not complete the study, 12 withdrew voluntarily and one was withdrawn due to noncompliance with the protocol.

**Table 1:** Baseline demographic characteristics of the study cohort.

Parameter		Indonesia	Malaysia	Philippines	Thailand	Vietnam	All Countries
N (%)		450 (100.0)	300 (100.0)	300 (100.0)	300 (100.0)	150 (100.0)	1,500 (100.0)
**Sex**	Male	226 (50.2)	156 (52.0)	147 (49.0)	144 (48.0)	69 (46.0)	742 (49.5)
	Female	224 (49.8)	144 (48.0)	153 (51.0)	156 (52.0)	81 (54.0)	758 (50.5)
**Age (years)**	Mean	7.8	8.6	8.2	9.4	8.7	8.4
	Median	8.0	9.2	8.2	10.0	8.5	8.5
	SD	2.62	2.94	3.56	3.50	2.65	3.12
	Range	2.0–14.3	2.0–13.9	2.1–14.8	2.4–15.0†	2.2–13.7	2.0–15.0†

† Where a participant was enrolled the day before his/her birthday, the age was rounded to 15.0 years. N is the number of participants present at Visit 1.

The overall study duration was 294 days (9.8 months): 285, 294, 233, 244 and 292 days in Indonesia, Malaysia, Philippines, Thailand and Vietnam, respectively. Participants were followed up for a mean of 237 days (7.9 months), ranging at the different study sites from 211 days (at Cebu, Philippines) to 277 days (at My Tho, Vietnam).

All the acute febrile participants presented to their identified healthcare facility for an acute visit. Overall, 96.5% presented within 5 days after fever onset (one participant [0.4%] presented initially to a non-study site, so the acute sample was taken outside the 5-day timeframe) and 96.9% returned within the designated period to have their convalescent blood sample drawn.

### Incidence and diagnosis of acute febrile episodes

The incidence density of acute fever overall was 33.6 (95% CI: 30.0; 37.8) per 100 person-years of follow-up, ranging from 20.8 in Malaysia to 40.5 in Indonesia (Table 2). Overall, 19.3% (289/1,500) of the cohort experienced at least one acute febrile episode during the study period (Table 2). A total of 374 acute febrile episodes occurred in 289 participants – 60 of these participants had two or more acute febrile episodes during the study period. Of these 60 participants, 20 reported three or more acute febrile episodes, three had four febrile episodes and one participant had five febrile episodes.

**Table 2:** Proportion and incidence density of acute febrile episodes in the study cohort.

Countries	N	Participants who had at least one febrile episode†	Percentage of participants	Incidence density per 100 person-years (95% CI)	Number of acute febrile episodes of any cause
**All countries**	1,500	289	19.3	33.6 (30.0; 37.8)	374
**Indonesia**	450	105	23.3	40.5 (33.5; 49.1)	137
**Malaysia**	300	38	12.7	20.8 (15.1; 28.5)	44
**Philippines**	300	61	20.3	39.2 (30.5; 50.4)	88
**Thailand**	300	53	17.7	32.4 (24.8; 42.5)	70
**Vietnam**	150	32	21.3	32.8 (23.2; 46.3)	35

† For each participant, only the first occurrence of an acute febrile episode was used to calculate the incidence density.

A clinical diagnosis was reported for 98.9% of febrile episodes (370/374). The five most frequently made clinical diagnoses using the Medical Dictionary for Regulatory Activities (MedDRA) preferred terms were: pharyngitis including nasopharyngitis: 124/374 acute febrile episodes (33%); upper and lower respiratory tract infections including upper respiratory tract infections, pneumonia, bronchitis: 72/374 (19%); tonsillitis including pharyngotonsillitis: 39/374 (10.5%); viral infection excluding dengue: 37/374 (10%); dengue: 34/374 (9.1%); and gastroenteritis including diarrhea: 8/374 (2%). Because of the length of time it took to process the laboratory tests, clinical diagnoses were often made independently of the reported laboratory results within the study context, and acute febrile participants were managed according to local standard practice.

A laboratory test result (i.e. laboratory diagnosis) for dengue, chikungunya, hepatitis A, influenza A, leptospirosis, rickettsia, and/or *S.* Typhi was reported for 95.7% (358/374) of febrile episodes (97.8% in Indonesia, 86.4% in Malaysia, 96.6% in the Philippines, 95.7% in Thailand and 97.1% in Vietnam). Overall, 57% of participants tested positive by IgM for one of these seven etiological agents.

### Incidence and diagnosis of dengue as the cause of acute febrile illness

**Figure 1:**
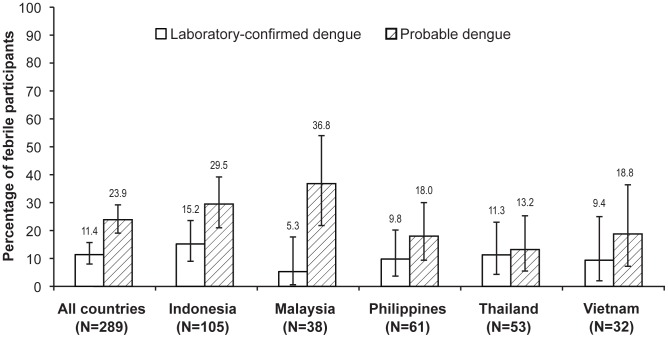
Percentage of virologically and serologically diagnosed dengue cases amongst participants who had at least one acute febrile episode during the study. Laboratory-confirmed dengue: NS1 antigen positive; Probable dengue: IgM positive and/or fourfold rise in IgG.

The overall incidence density of laboratory-confirmed dengue by NS1 antigen was 3.4 (95% CI: 2.4; 4.8) per 100 person-years, and of probable dengue by serology was 7.3 (95% CI: 5.7; 9.2) per 100 person-years (Table 3). The mean duration of fever at the time of blood sampling for NS1 testing was 2 days (median 2.4 days). Of the 289 febrile participants, 11.4% (95% CI: 8.0; 15.7) had laboratory-confirmed dengue, while 23.9% (95% CI: 19.1; 29.2) had probable dengue (Figure 1).

**Table 3:** Incidence density of dengue in febrile participants.

Countries (N)	Laboratory-confirmed dengue		Probable dengue	
	Cases†	Incidence density (95% CI)‡	Cases†	Incidence density (95% CI)‡
**All countries (1,500)**	33	3.4 (2.4; 4.8)	69	7.3 (5.7; 9.2)
**Indonesia (450)**	16	5.3 (3.3; 8.7)	31	10.5 (7.4; 15.0)
**Malaysia (300)**	2	1.0 (0.3; 4.1)	14	7.3 (4.3; 12.4)
**Philippines (300)**	6	3.4 (1.5; 7.7)	11	6.4 (3.5; 11.5)
**Thailand (300)**	6	3.3 (1.5; 7.3)	7	3.9 (1.8; 8.1)
**Vietnam (150)**	3	2.7 (0.9; 8.3)	6	5.4 (2.4; 12.0)

† For each participant, only the first occurrence of a dengue-positive acute febrile episode was used to calculate incidence density.

‡ Incidence density per 100 person-years of study follow-up. Laboratory-confirmed dengue: NS1 positive; Probable dengue: IgM positive and/or fourfold rise in IgG.

Discrepancies between clinical diagnosis of dengue and laboratory test findings were observed. As mentioned previously, a clinical diagnosis of dengue was made in 34 out of 374 febrile episodes (9.1%). Sixteen per cent (60/374) of acute febrile episodes were not clinically diagnosed as dengue but were supported by serology (i.e. probable dengue), and 5.3% (20/374) were not clinically diagnosed but were supported by virological testing (i.e. laboratory-confirmed dengue). By contrast, 5.9% of acute febrile episodes (22/374) were clinically diagnosed as dengue, but were not supported by the laboratory tests for NS1 and/or or IgM/IgG.

### Other causes of acute febrile illness

**Figure 2:**
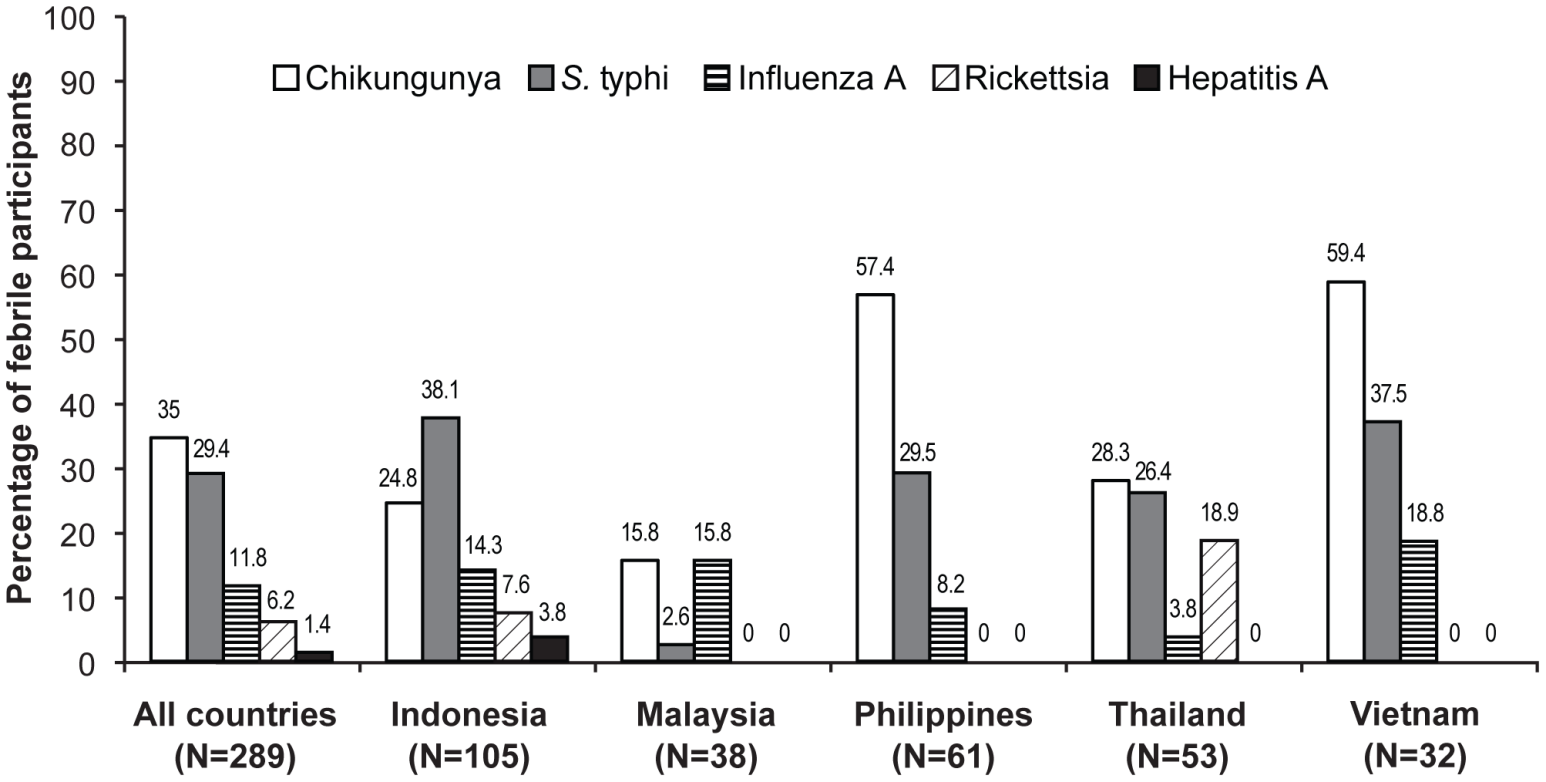
Frequency of most commonly detected non-dengue infections in febrile participants. Data are the percentage of participants who had at least one acute febrile episode during the study, for whom IgM antibodies to these pre-specified infections were detected in acute or convalescent sera.

Of the prespecified panel of non-dengue diseases for which sera from acute febrile participants were tested, only chikungunya and typhoid fever were laboratory-diagnosed by IgM positivity at incidence densities that were higher than that of dengue (10.8 [95% CI: 8.9; 13.1] and 9.1 [95% CI: 7.3; 11.2] per 100 person-years, respectively; Table 4). Chikungunya virus IgM antibodies were detected in 35.0% of febrile participants, and typhoid fever IgM antibodies in 29.4% (Figure 2).

**Table 4:** Incidence density of non-dengue infections in febrile participants.

Country	Chikungunya		Typhoid fever		Influenza A		Rickettsia		Hepatitis A	
	Cases†	Incidence density‡ (95% CI)	Cases†	Incidence density‡ (95% CI)	Cases†	Incidence density‡ (95% CI)	Cases†	Incidence density‡ (95% CI)	Cases†	Incidence density‡ (95% CI)
**All countries**	101	10.8 (8.9; 13.1)	85	9.1 (7.3; 11.2)	34	3.5 (2.5; 5.0)	18	1.9 (1.2; 3.0)	4	0.4 (0.2; 1.1)
**Indonesia**	26	8.8 (6.0; 12.9)	40	13.9 (10.2; 19.0)	15	5.0 (3.0; 8.3)	8	2.7 (1.3; 5.3)	4	1.3 (0.5; 3.5)
**Malaysia**	6	3.1 (1.4; 6.9)	1	0.5 (0.1; 3.6)	6	3.1 (1.4; 6.9)	0	0 (NC)	0	0 (NC)
**Philippines**	35	21.3 (15.3; 29.6)	18	10.5 (6.6; 16.7)	5	2.9 (1.2; 6.9)	0	0 (NC)	0	0 (NC)
**Thailand**	15	8.5 (5.1; 14.1)	14	7.9 (4.7; 13.4)	2	1.1 (0.3; 4.4)	10	5.5 (3.0; 10.3)	0	0 (NC)
**Vietnam**	19	18.5 (11.6; 28.6)	12	11.1 (6.3; 19.6)	6	5.4 (2.4; 12.1)	0	0 (NC)	0	0 (NC)

Acute infections were determined by IgM positivity.

† For each participant, only the first occurrence of an infection was used to calculate incidence density.

‡ Incidence density per 100 person-years of follow-up. NC: not calculated.

Influenza, rickettsia and hepatitis A were less common causes of febrile illness than dengue. Three cases of leptospirosis were detected by hemagglutination (one in the Philippines and two in Thailand). However, this testing method does not allow one to distinguish between IgM and IgG antibodies, and therefore it could not be confirmed whether leptospirosis was the cause of these acute febrile episodes, or whether IgG antibodies remained from a previous infection.

Amongst the 218 febrile participants who tested negative for dengue, there were 82 laboratory-diagnosed cases of chikungunya (i.e. 28.4% of all febrile participants), 65 cases of typhoid fever (22.5%), 21 cases of influenza A (7.3%), 10 cases of rickettsia (3.5%) and 4 cases of hepatitis A (1.4%). Among the 71 febrile participants who tested positive for dengue by NS1 antigen and/or serology, there were 17 laboratory-diagnosed cases each of chikungunya and typhoid fever (each 5.9% of all febrile participants), 13 cases of influenza A (4.5%) and 7 cases of rickettsia (2.4%).

## Discussion

This is the first prospective, multinational, active surveillance study with a focus on acute febrile illness to include these five dengue-endemic Asian countries. The overall incidence density of acute febrile illness was 33.6 per 100 person-years, with 19.3% of the 1,500 children experiencing at least one episode. This proportion is close to the estimated rate of 24% that was anticipated at the time of the study design, based on unpublished national and regional reports. Overall, 57% of participants with acute febrile illness tested positive by IgM for one of the seven etiological agents included in the predetermined panel of commercial tests.

The incidence density of dengue was 3.4 per 100 person-years according to NS1 antigen positivity (11.4% of febrile participants) and 7.3 by serology (23.9% of febrile participants), which confirms the high dengue endemicity in these countries. Where these findings differed from those reported previously for other dengue surveillance studies in this region, it was most likely because dengue incidences can vary from year to year, even at the same site [2], [5], [7], [17], [24], [25]. For example, in an active surveillance study of acute febrile illness among school children in Ratchaburi, Thailand from 2006 to 2009, dengue (confirmed by IgM/IgG ELISA) caused 6.74% of all acute febrile illnesses during the total study period, and the incidence ranged from 1.77% in 2006 to 5.74% in 2008 [5]. Another active surveillance study among children in Ratchaburi and Kamphaeng Phet reported dengue incidences of 23–25/1,000 in 2006–2007 using IgM/IgG and/or RT-PCR or virus isolation [17]. Incidence rates of dengue ranged from 16.9 to 38.6 per 1,000 person-years following active surveillance of 2–15-year-olds in Long Xuyen, Vietnam from 2003 to 2007 [25]. In the Philippines, a surveillance study conducted in San Pablo from 2007 to 2009 showed that 11% of acute febrile illnesses in infants were caused by dengue [26].

The proportion of dengue cases confirmed by serology was greater than those confirmed by NS1 antigen. Several explanations could account for this. Sensitivity of NS1 testing is directly related to the viral load, and thus the time since the start of viral replication: the mean duration of fever at the time of sampling was 2.4 days. Although the NS1 antigen test used in this study to test for laboratory-confirmed dengue has high specificity [18], [27], DENV-2 infections have been associated with significantly lower plasma NS1 levels relative to DENV-1 or DENV-3 infections [28]. In addition, a lower sensitivity for this test has been reported for secondary infections and, in dengue-endemic areas (such as those where this study was conducted), a higher proportion of patients have secondary infections [27]. Differences in primary versus secondary dengue infections and non-dengue Flavivirus prevalences may have influenced the serology findings as follows. Firstly, IgM antibodies for dengue can remain elevated for 2–3 months after infection [29], and positive IgM results could have been recorded for samples where an infection occurred 2–3 months before acute sample collection. Secondly, serological kinetics differ between primary and secondary dengue infections [30]. In secondary dengue infections, IgM levels are substantially lower than in primary infections [30], while IgG antibody titers are detectable even in the acute phase, and rise rapidly and cross-react broadly with other Flaviviruses [29]. The differences in findings between methods may thus be due in part to variabilities in dengue epidemiology, seasonality and serotype prevalence between the study countries [31]. However, this effect could not be assessed directly because of this study's limitations that neither seroprevalence (i.e. dengue serotypes) nor primary versus secondary infections were determined.

That chikungunya was the most commonly detected infection of those selected for evaluation in this study, occurring in a relatively high proportion (35.0%) of acute febrile participants with an incidence density of 10.8 per 100 person-years, was rather unexpected. However, even allowing for the possibility that chikungunya-specific IgM levels can remain elevated for up to 60 days [32], these findings are consistent with reports that chikungunya has re-emerged as an important infection in Asia [8], [33]–[35]. The high incidence density of chikungunya observed in the Philippines (21.3) and Vietnam (18.5 per 100 person-years), which contributed to the high incidence observed overall, are consistent with local news and internet reports that chikungunya cases increased before or during the study period in these countries (unpublished). Furthermore, a surveillance study of chikungunya in children aged <15 years, conducted in southern Vietnam in 2010 in a region that included our study site, showed that 0–33.3% of sera samples were positive for chikungunya at different sites [36]. Two chikungunya outbreaks were also reported in the region during the study period: one in China from September to October 2010 [37], and 1,500 cases of chikungunya were reported in Cambodia between May 2011 and June 2012 [38]. Although studies that track the incidence of chikungunya during outbreaks have been carried out in southern Thailand [8], [35], [39] and Indonesia [40], this is the first report to describe the incidence of chikungunya in these five countries following active surveillance.

Typhoid fever was the second most commonly detected infection overall, being identified in 29.4% of febrile participants at an incidence density of 9.1 per 100 person years. This finding must be viewed in the context of the commercial test's limited sensitivity and specificity (discussed further below). Nevertheless, our findings suggest that implementing routine tests for chikungunya (namely, antigen detection) and typhoid fever (culture) in these countries would increase the accuracy of diagnosis of undifferentiated acute febrile illness in children. Influenza vaccination is mainly used in the private market in these countries, hence the rates of influenza vaccination were most likely low in the study population (which was defined as healthy children at the time of enrolment), although these data were not collected from participants.

Although the commercial kits used for the pre-specified panel of non-dengue diseases were not the gold standard tests for some of these infectious agents, they were used because standardization was essential in this multicountry study and they provided ease of use. To eliminate confounding factors where positive serology results could have been indicative of previous infections or cross-reactivity, we reported only the IgM data for non-dengue causes of acute infections because IgG data (and thus IgM/IgG ratios) were not available for all the causative infectious agents. Nevertheless, we acknowledge the limitations of using these test results to calculate the incidences of these causative agents without correlating the findings with additional more stringent tests. The chikungunya test that was used has ≥95% sensitivity and specificity and, according to the manufacturer's specifications, does not cross-react with dengue antibodies (Table S1). However, test performances based on manufacturers' data may be higher than those based on published data, which generally include many more samples per study. Hence, it is possible that testing in the absence of correlation with clinical diagnoses could have led to an overestimation of the true disease incidence of chikungunya, although internet-based news bulletins such as the Program for Monitoring Emerging Diseases (ProMED-mail, a programme of the International Society for Infectious Diseases) confirm that chikungunya cases are increasing in these countries.

The limitations of rapid testing for *S.* Typhi using currently available commercial tests have been well debated in the literature [3], [41]–[43]. Blood culture from blood or bone marrow and microbiological characterization are the gold standard of enteric fever diagnosis, yet even these methods are only positive in 40%–60% of presumptive cases [41]. Despite the increased sensitivity of bone marrow culture, obtaining bone marrow by standard methods is technically challenging, invasive and not generally performed. In view of the importance of this pathogen in the highly endemic region of Southeast Asia [3], [44], we opted to use a commercialized test in order to try to capture as much information on *S.* Typhi infection as possible. The sensitivity and specificity of the *S.* Typhi IgM ELISA test we used were calculated from manufacturer's product information to be 86% and 96%, respectively (Table S1). In addition, a larger proportion of *S.* Typhi-positive participants than those positive for the other agents were IgM-positive for other causative organisms. Our findings should thus be viewed in the context of these factors and are likely to be an overestimation of the true incidence of *S.* Typhi as a causative agent of acute febrile disease.

Discrepancies between clinical diagnoses and laboratory confirmation of dengue infection were observed. Dengue infections were clinically underdiagnosed: 16.0% of acute febrile episodes that were not clinically diagnosed as dengue were later supported by positive dengue serology, and 5.3% were confirmed by NS1 antigen testing. Clinical misdiagnoses were also made: 5.6% of all acute febrile episodes were clinically diagnosed as dengue but were not laboratory-confirmed. These findings confirm previous reports from this region [4], [16] that clinical diagnosis of dengue has a limited predictive value and that laboratory analysis supports a more accurate assessment in differentiating causes of fever. Such discrepancies impact the accuracy of disease burden estimates.

A study limitation is that the disease incidences that we report here are based on laboratory test findings alone, and may represent a misestimation of the true incidence of some of these etiological causes of febrile illness, in the context of the specificity and sensitivity of each of these respective tests. Clinical diagnosis was not taken into account when calculating disease incidence, except for dengue. Nevertheless, a strength of this study was its prospective cohort design involving intensive active surveillance to capture cases that might not otherwise have been detected on the basis of symptoms alone. Acting to ‘correct’ these incidences by excluding participants with atypical presentation would have compromised this study strength. The limited sample size (150 children per center) and the relatively short duration of less than 1 year is another study limitation. Nevertheless, given that a Phase III efficacy study of a dengue vaccine commenced at these sites immediately following this study (ClinicalTrials.gov NCT01373821), these data provided valuable information about the baseline incidence of acute febrile illness and dengue. The study also met its secondary objective of showing that all these sites were capable of capturing and following up acute febrile episodes within a specific timeframe among a well-defined cohort, which lends additional validity to the data presented here.

In conclusion, active fever surveillance showed that of the seven diseases for which we tested, the most common causes of pediatric acute febrile illness in these countries were chikungunya, *S.* Typhi and dengue. Clinical diagnosis was not sufficient to detect all dengue cases, and laboratory confirmation is essential to refine disease burden estimates of dengue and other common causes of acute febrile illness in children. These findings are of relevance to researchers planning clinical studies of vaccines against these infectious agents in Southeast Asia.

## Supporting Information

Checklist S1 **STROBE statement.** Checklist of items included in this cohort study.
(DOC)

Table S1 **Sensitivity and specificity of the commercial laboratory test kits used to test sera for non-dengue causes of febrile illness in this study.**
(DOC)
