# Supplementary material for: Dengue and Other Common Causes of Acute Febrile Illness in Asia: An Active Surveillance Study in Children
Source: PLoS Negl Trop Dis. 2013 Jul 25;7(7):e2331. doi: 10.1371/journal.pntd.0002331 (PMC3723539; doi:10.1371/journal.pntd.0002331)
Supplement: Table S1 — Sensitivity and specificity of the commercial laboratory test kits used to test sera for non-dengue causes of febrile illness in this study. (DOC) [file pntd.0002331.s002.doc]

**Table S1.** Sensitivity and specificity of the commercial laboratory test kits used to test sera for non-dengue causes of febrile illness in this study

| **Kit (Manufacturer)** | **Used to detect** | **Sensitivity** | **Specificity** | **Cross-reactivity** | **Source of information** |
| --- | --- | --- | --- | --- | --- |
| **NovaLisa Chikungunya IgM μ-capture ELISA** (NovaTec Immundiagnostica GmbH, Germany) | Chikungunya-specific IgM antibodies | 95.5 % | 100% | No cross-reactivity was observed by using Rheumatoid factor-samples and samples containing antibodies against *Bordetella pertussis*, *Chlamydia trachomatis*, *Chlamydia pneumoniae*, Dengue Virus, TBE, *Helicobacter pylori*, HSV 2, Leishmania, Mycoplasma and Schistosoma. Cross reactivity with antibodies against *Borrelia*, CMV and Toxoplasma and other alpha viruses cannot be excluded | Product information (available at http://www.novatec-id.com/fileadmin/user_upload/Flyers/Flyer_-_Novalisa_-_English_-_0311_01.pdf) and additional information provided by manufacturer on request |
| ***Salmonella* Typhi IgM ELISA** (Calbiotech Inc, USA) | *S*. Typhi-specific IgM antibodies | ~ 86% | 96% | Not stated in product information; manufacturer did not respond to requests for further information | Calculated from information on Kit package insert (available at: http://www.calbiotech.com/images/stories/virtuemart/product/st084m-r3-rc.pdf) |
| **Anti-HAV IgM ELISA** (DIAsource ImmunoAssays S.A., Belgium) | IgM antibody to hepatitis A virus | 100% | 100% | No cross-reactivity against hepatitis B | Product information (available at: http://www.diasource-diagnostics.com/var/ftp_diasource/IFO/KAPG4AME3.pdf) |
| **NovaLisa Influenza Virus A IgM-ELISA** (NovaTec Immundiagnostica GmbH, Germany) | IgM antibodies to influenza virus A | >95% | >95% | Not stated in package insert | Product information (available at: http://www.novatec-id.com/fileadmin/user_upload/Flyers/Flyer_-_Novalisa_-_English_-_0311_01.pdf) |
| **Rickettsia IFA IgM** (Focus Diagnostics, USA) | IgM antibodies to Spotted Fever and Typhus Fever group Rickettsia | The Focus Diagnostics Rickettsia IFA IgM kit was compared with both IFA and CF tests from the Centers for Disease Control (CDC) using a panel of 32 coded sera. The IgM test demonstrated 100% correlation of positives, negatives, and endpoint titer (within 1 serial dilution) when the IFA procedure was compared both for Spotted Fever group and Typhus Fever group. There was also complete correlation for positives and negatives compared with the CDC CF test. | | Cross-reactivity within the Spotted Fever group or the Typhus Fever group precludes the speciation of the infecting Rickettsia by this procedure. Sera reactive with *R. rickettsii* must be termed “Spotted Fever group positive”, while those reactive with *R. typhi* are termed “Typhus Fever group positive.” | Product information (available at: http://www.focusdx.com/pdfs/pi/OUS/IF0100M.pdf)  Reference citing this kit: |
| **Leptospirosis Indirect Hemagglutination (IHA) Test** (Focus Diagnostics, USA) | Group-specific antibodies to Leptospira | 100% | | 97% | Product information (available at: http://www.focusdx.com/pdfs/pi/OUS/IH0100.pdf) |
|  |  | 92%, | | 95% |  |

**References:**

1. Chapman AS, Bakken JS, Folk SM, Paddock CD, Bloch KC, et al. (2006) Diagnosis and management of tickborne rickettsial diseases: Rocky Mountain spotted fever, ehrlichioses, and anaplasmosis--United States: a practical guide for physicians and other health-care and public health professionals. MMWR Recomm Rep 55: 1–27.

2. Sulzer CR, Glosser JW, Rogers F, Jones WL, Frix M (1975) Evaluation of an indirect hemagglutination test for the diagnosis of human leptospirosis. J Clin Microbiol 2: 218–221.
